# Chicago Medical Society, August 3, 1874

**Published:** 1874-09-01

**Authors:** Will. T. Montgomery


### CHICAGO MEDICAL SOCIETY.

REGULAR SEMI-MONTHLY MEETING, AUG 3, 1874.

*Reported by Will. T. Montgomery, M.D.*

DR. QUINE read a paper presenting the most important facts from a record of a number of cases of puerperal metritis, metro-peritonitis and puerperal septicemia, which had occured in Cook County Hospital. In the cases presented, the line of distinction between the three diseases was pretty clearly drawn. With reference to treatment, blisters and poultices to the abdomen were used in all cases. The chief treatment in the cases of simple metritis was veratrum or aconite, combined with opium sufficient to relieve pain; and attention to cleanliness. The cases of puerperal septicemia were treated substantially as typhoid fever. The opium treatment was used in the cases of metro-peritonitis. In two cases that recovered, half drachm doses of morphine were given for several days in succession. The pulse was kept between four and eight in the minute. To one patient seven grains of morphia were given several successive hours, and for two days previously five grains were administered every hour. The morphine was gradually withdrawn three days before death, and while it was used no effect was noticed on the pulse or respiration. Veratrum was faithfully tried, but invariably increased the frequency and diminished the fullness of the pulse. Quinine had a marked effect in diminishing the intensity of the fever. His experience was decidedly in favor of opium and quinine.

Discussion—Dr. C. M. Fitch had seen five well marked cases of puerperal fever, and all were fatal. He related a case following abortion which forcibly impressed him with the contagiousness of the disease. All he had read on the subject had been eminently unsatisfactory. Dr. Jacobson had seen local depletion, by means of leeches to the abdomen used a great deal, but had not been encouraged to use it in his own practice. He had seen good effect from the use of collodion to the abdomen. There is a great difference in regard to prognosis, in different epidemics. Little can be done after the disease is well established. We cannot exercise too much care in the prevention by contagion. He thought the disease often arose from products of the uterus, retained by inertia, and recommended the use of ergot in cases of faulty contractions. Two cases he had seen in which the death of the fœtus seemed to have been the cause of the disease. Dr. T. D. Fitch had had but little experience with the disease in private practice. He agreed with Dr. Jacobson in reference to the importance of prophylaxis. Patients may be infected by the physician after making autopsies, or attending erysipelatous cases. The greatest care should be observed in disinfecting the hands and clothing. He coincided with the views of Dr. Jacobson with regard to the retained products of the uterus and ergot. He is particular to give ergot in all cases in which there had previously been post partum hemorrhage or inertia. Injury to the parts may be an exciting cause of the disease. As regards mortality, much depends upon the severity of the epidemic. In the treatment of puerperal cases he secures an early evacuation of the bowels—prefers castor oil. He did not use vaginal injections unless the discharges were offensive. He usually began the treatment of the fever by giving veratrum in four-drop doses, and increased the dose until the pulse was brought down to sixty or seventy. In malignant cases, veratrum would not reduce the pulse, and stimulants were indicated. He used blisters, but never bleeding, and withdrew the veratrum as the case progressed. Heroic doses of opium were less to be advised than those sufficient to control pain. Dr. Stillians had never had a case of fever in a patient which he had delivered, and had flattered himself that it was due to his management of his patients. He used vaginal injections in all his labor cases. Dr. Strong had seen eight cases; six died and two recovered. One of the cases that recovered was leeched, and in the other, stimulants and quinine were used. Dr. Paoli said that we labor, under the delusion that these cases sometimes recover, he did not believe any well marked cases ever recovered, and he did not believe any gentleman would aver that he had seen such recover. He did not believe it was always possible to distinguish between the different puerperal diseases. Bleeding and veratrum do not have any curative effect, but only control the severity of the symptoms; so with quinine. No medicines had any curative effect in this disease. A thorough examination will always discover pus in the blood of the puerperal fever cases. Dr. Millard had never been able to distinguish between puerperal fever and peritonitis; he had had success from venesection in the treatment of these cases. He considered complete contraction of the uterus after delivery most important.

Dr. Taggart had seen the veratrum and opium treatment used in an epidemic in Buffalo, with unsatisfactory results. Dr. Van Buren did not know what puerperal fever was; he had been taught from books that it is an inflammation; if we knew just what it is we might treat it intelligently; if there was any truth in the poison theory, he thought it was important to secure firm contractions of the uterus; he had never seen but one case of the fever, and that died. Dr. Etheridge inquired as to the large doses administered in one of the cases reported. Did the patient really take seven grains of morphia for a number of hours in succession? Dr Quine responded that the dose was gradually increased to seven grains, and repeated a number of times; such heroic doses were only administered in the one case. Some important points had been brought out in the discussion, that were not alluded to in the paper; first, as to the contagiousness and nature of the disease. He had used the term generically, and did not think there was any connection between puerperal fever and other inflammations; the poison may enter the system through any open blood vessels. Puerperal fever might occur before confinement. He had never communicated the disease, and latterly had not used great care. The specific virus may be readily communicated by one, and not by another. One midwife had recently furnished him eleven malignant cases.

He had seen cases in which the injudicious use of cathartics seemed to aggravate the disease, and hasten a fatal termination. He had used quinine and nux vomica in cases of inertia with good effect. Quinine was the most efficient remedy in restraining the disease, and he administered it to cinchonism. He had also used stimulants in most cases. Pain was not a marked symptom, and he did not give opium for its anodyne, but for its specific, effect upon the inflammation. He was as sure as he could be without autopsy, that he had seen true cases of puerperal fever recover.

Dr. Mary Thompson asked: Is it right for a physician to go from a case of eruptive fever to a case of confinement?

Dr. Paoli answered in his usual positive manner, "No!" After miscellaneous business was disposed of, a motion to adjourn prevailed.

